# A Phosphorylation-Induced Switch in the Nuclear Localization Sequence of the Intrinsically Disordered NUPR1 Hampers Binding to Importin

**DOI:** 10.3390/biom10091313

**Published:** 2020-09-11

**Authors:** José L. Neira, Bruno Rizzuti, Ana Jiménez-Alesanco, Martina Palomino-Schätzlein, Olga Abián, Adrián Velázquez-Campoy, Juan L. Iovanna

**Affiliations:** 1 Instituto de Biología Molecular y Celular, Universidad Miguel Hernández, 03202 Elche, Spain; 2 Instituto de Biocomputación y Física de Sistemas Complejos (BIFI), Joint Units IQFR-CSIC-BIFI, and GBsC-CSIC-BIFI, Universidad de Zaragoza, 50009 Zaragoza, Spain; ajimenez@bifi.es (A.J.-A.); oabifra@unizar.es (O.A.); adrianvc@unizar.es (A.V.-C.); 3 CNR-NANOTEC, Licryl-UOS Cosenza and CEMIF.Cal, Department of Physics, University of Calabria, Via P. Bucci, Cubo 31 C, 87036 Arcavacata di Rende, Cosenza, Italy; bruno.rizzuti@cnr.it; 4 Centro de Investigación Príncipe Felipe, 41930 Valencia, Spain; martina@tinet.org; 5 Instituto de Investigación Sanitaria Aragón (IIS Aragón), 50009 Zaragoza, Spain; 6 Centro de Investigación Biomédica en Red en el Área Temática de Enfermedades Hepáticas y Digestivas (CIBERehd), 28029 Madrid, Spain; 7 Departamento de Bioquímica y Biología Molecular y Celular, Universidad de Zaragoza, 50009 Zaragoza, Spain; 8 Instituto Aragonés de Ciencias de la Salud (IACS), 50009 Zaragoza, Spain; 9 Fundación ARAID, Gobierno de Aragón, 50009 Zaragoza, Spain; 10 Centre de Recherche en Cancérologie de Marseille (CRCM), INSERM U1068, CNRS UMR 7258, Aix-Marseille Université and Institut Paoli-Calmettes, Parc Scientifique et Technologique de Luminy, 163 Avenue de Luminy, 13288 Marseille, France

**Keywords:** circular dichroism, flexibility, fluorescence, importin, intrinsically disordered protein, isothermal titration calorimetry (ITC), molecular docking, nuclear magnetic resonance (NMR), nuclear protein 1 (NPR1), peptide

## Abstract

Several carrier proteins are involved in protein transport from the cytoplasm to the nucleus in eukaryotic cells. One of those is importin α, of which there are several human isoforms; among them, importin α3 (Impα3) has a high flexibility. The protein NUPR1, a nuclear protein involved in the cell-stress response and cell cycle regulation, is an intrinsically disordered protein (IDP) that has a nuclear localization sequence (NLS) to allow for nuclear translocation. NUPR1 does localize through the whole cell. In this work, we studied the affinity of the isolated wild-type NLS region (residues 54–74) of NUPR1 towards Impα3 and several mutants of the NLS region by using several biophysical techniques and molecular docking approaches. The NLS region of NUPR1 interacted with Impα3, opening the way to model the nuclear translocation of disordered proteins. All the isolated NLS peptides were disordered. They bound to Impα3 with low micromolar affinity (1.7–27 μM). Binding was hampered by removal of either Lys65 or Lys69 residues, indicating that positive charges were important; furthermore, binding decreased when Thr68 was phosphorylated. The peptide phosphorylated at Thr68, as well as four phospho-mimetic peptides (all containing the Thr68Glu mutation), showed the presence of a sequential NN(*i*,*i* + 1) nuclear Overhauser effect (NOE) in the 2D-^1^H-NMR (two-dimensional–proton NMR) spectra, indicating the presence of turn-like conformations. Thus, the phosphorylation of Thr68 modulates the binding of NUPR1 to Impα3 by a conformational, entropy-driven switch from a random-coil conformation to a turn-like structure.

## 1. Introduction

Active nuclear translocation happens through importins (also known as karyopherins), together with other proteins such as the GTPase Ran and nucleoporins [1,2,3]. The classical nuclear import pathway is started by recognition of a nuclear localization sequence (NLS) in the cargo by importin α [4]. The complex cargo importin α binds to importin β; then, this complex goes through the nuclear pore complex (NPC). The GTPase Ran dissociates the ternary complex within the nucleus by interacting with importin β, and both importins α and β are recycled back to the cytoplasm [4]. The human genome encodes seven isoforms of importin α, with three subtypes [4,5,6]. These isoforms have a role in cell differentiation, gene regulation [5,7], and even in viral infections, because some viral proteins are recognized by specific importins [8].

Importin α is a modular protein built of α-helix repeat armadillo (ARM) units [1,4]. It has two domains: (i) a N-terminal importin β-binding (IBB) domain, approximately 60-residues-long, which is used for binding to importin β before transport through the NPC, and (ii) a C-terminal NLS-binding motif formed by ten ARM units [9]. Structures of several truncated importin α, without the IBB domain [8,9], have shown that the cargo NLS region binds in a disordered conformation. This interaction occurs at a concave site of the elongated structure, involving ARM motifs 2 to 4 (major site) or 6 to 8 (minor site) for the shortest classical monopartite NLSs or both sets of ARM motifs for the largest bipartite NLS regions. When importin β is not present, the IBB domain, which mimics an NLS region, occupies the ARM motifs involved in NLS recognition [9]. This intramolecular interaction has an autoinhibitory role, and it is thought to be relevant in cargo dissociation in the nucleoplasmic side [9].

Intrinsically disordered proteins (IDPs) do not have a unique stable conformation, resulting in a dynamic conformational ensemble that is reflected in a high structural flexibility. They are involved in cell cycle control, signaling, molecular recognition, replication, and transcription processes [10,11,12,13]. The discovery of IDPs has shown that protein biological activity is possible even without a well-defined structure [12,13,14] but, rather, with an extreme structural flexibility. However, IDPs may have a propensity to adopt structures at the local level; this acquisition of local order can be achieved by, among other factors, post-translational modifications [14]. Such modifications, in turn, can widen their biological functions [11,15]. NUPR1 (UniProtKB O60356) is an 82-residue-long (8 kDa), highly basic, monomeric IDP that is overexpressed during the acute phase of pancreatitis [16,17] and in almost any, if not all, cancer tissues [18]. Its exact functions are unknown, but NUPR1 is a key element in the cell-stress response and cell-cycle regulation [18,19]. Moreover, NUPR1 intervenes in apoptosis through the formation of a complex with the oncoprotein ProTα [20] and in DNA repair [21,22]. In the interactions with all these partners and other synthetic molecules, NUPR1 uses two hotspots around residues Ala33 and Thr68 [22,23,24]. In addition, NUPR1 has a bipartite NLS region around Thr68, which is fully functional [25]. Thus, even though NUPR1 is a relatively small protein, it might require the assistance of the importin system for nuclear translocation due to its unfolded nature and its large radius of gyration, which would be closer to the limit of free diffusion through the NPC. In addition, NUPR1 might require the presence of importins to avoid undesired interactions with other macromolecules in the cytoplasm, due to its basic nature [26].

In this work, we have studied the interaction of human importin α3 (Impα3), also called KPNA4, and that of its truncated species, without the IBB domain (ΔImpα3), to either NUPR1 or peptides encompassing its NLS (NLS-NUPR1). We have chosen Impα3 as a target for NUPR1 because of its larger flexibility when compared with other importins, as concluded by the structural factors from the X-ray data, which confers in it a greater ability to interact with cargos, having a higher variety of conformations [8]. From an experimental pint of view, Impα3 can be also easily expressed and purified for in vitro structural studies [8]. Interestingly, it has also been shown to be crucial in pain pathways [27]. In addition, by studying both importin species (with and without the IBB), we were interested in finding out whether the absence of the IBB domain affected the binding of NLS-NUPR1. The NLS-NUPR1 peptides had mutations at: (i) the two lysines in the sequence (Lys65 and Lys69), which are important for nuclear translocation, according to in vivo studies [25], and (ii) Thr68, where we have either introduced phospho-threonine or, alternatively, we have designed phospho-mimetic mutations (with a glutamic residue). We have used several spectroscopic and biophysical techniques—namely, steady-state fluorescence, circular dichroism (CD), nuclear magnetic resonance (NMR), isothermal titration calorimetry (ITC), and molecular docking—to address the binding of the peptides to both importins. Our results indicate that the isolated wild-type (wt) NLS-NUPR1, as well as the mutants, were monomeric and disordered in the solution. The wt NLS-NUPR1 peptide bound to both importins, and the affinity was larger for ΔImpα3 (0.95 μM versus 1.7 μM for Impα3), indicating that the IBB region must have an inhibitory effect; this result is in agreement with other binding studies involving intact, well-folded protein cargos [9], but to the best of our knowledge, this is the first time tested with an IDP. The binding of NLS-NUPR1 peptides to both importins was hampered by removal of either Lys65 or Lys69, and it was almost abolished when Thr68 was phosphorylated or when the phospho-mimetics were assayed. Interestingly enough, the phosphorylated peptide at Thr68 and the four phospho-mimetics showed the presence of turn-like conformations, which were not observed in the wt NLS-NUPR1 peptide or in the Lys65Ala or Lys69Ala mutants. We concluded that the phosphorylation of Thr68 modulates the binding of NUPR1 to importin by a conformational switch from a random-coil to a turn-like conformation.

## 2. Materials and Methods

### 2.1. Materials

Isopropyl-β-d-1-tiogalactopyranoside and ampicillin were obtained from Apollo Scientific (Stockport, UK). Imidazole, kanamycin, Trizma base, and His-Select HF nickel resin were from Sigma-Aldrich (Madrid, Spain). Protein marker (PAGEmark Tricolor) and Triton X-100 were from VWR (Barcelona, Spain). Amicon centrifugal devices were from Millipore (Barcelona, Spain), and they had a cut-off molecular weight of 30 or 50 kDa. The rest of the materials were of analytical grade. Water was deionized and purified on a Millipore system.

### 2.2. Protein Expression and Purification

The His-tagged ΔImpα3 (residues 64-521) was obtained from BL21 (DE3) cells as described [8]. The DNA of the codon-optimized, intact Impα3 with a His-tag at the N terminus was synthesized by NZYtech (Lisbon, Portugal) and cloned into the pHTP1 vector (with kanamycin resistance). Expression and purification of Impα3 were carried out as those for ΔImpα3 in the same *Escherichia coli* strain. Concentration of both species was determined from their six tyrosines and six tryptophans [28].

### 2.3. Design and Synthesis of the Peptides

The peptides were synthesized by NZYtech with a purity of 95%. The peptides comprised the NLS region of NUPR1 (Table 1); peptides were named with the accompanying name within parenthesis for each sequence, as reported in Table 1. All peptides were acetylated and amidated at the N and C termini, respectively, to avoid fraying effects. As the wt NLS had no tyrosine, we introduced one at the N terminus to allow for absorbance measurements [28]. We synthesized eight peptides with different mutations, with the following rationale: (i) we studied the importance of positions Lys65 and Lys69 in the binding to both importins by mutating the two positions to alanine, (ii) we mutated Thr68 to the glutamic T68E peptide to have a phosphomimic at this position, (iii) we combined this mutation at Thr68 with either of the other two as double mutants, as well as to both in a triple mutant, and (iv) we designed the phosphorylated peptide at position Thr68 (pT68 peptide) to study the effects of this single post-translational modification.

### 2.4. Fluorescence

#### 2.4.1. Steady-State Fluorescence

Fluorescence spectra were collected on a Cary Varian spectrofluorometer (Agilent, Santa Clara, CA, USA) with a Peltier unit. The samples were prepared the day before and left overnight at 278 K; before experiments, samples were left for 1 h at 298 K. A 1-cm-pathlength quartz cell (Hellma, Kruibeke, Belgium) was used. Concentrations of the peptides were 10 μM and that of importins was 4 μM. Samples containing the isolated peptide, the isolated importin, and the mixture of both, at those concentrations, were prepared for each peptide and each importin. Experiments were acquired at pH 7.0 in 50-mM phosphate buffer.

Protein samples were excited at 280 and 295 nm (although the samples of the isolated peptides did not show any fluorescence at the latter value). The other experimental parameters and the buffers used have been described elsewhere [30]. Appropriate blank corrections were made in all spectra.

#### 2.4.2. Thermal Denaturations

Thermal denaturations were performed at 60 K/h with an average time of 1 s for all samples. Thermal scans were collected at 315, 330, and 350 nm after excitation at 280 or 295 nm from 298 to 358 K. The rest of the experimental set-up was the same as described above. Thermal denaturations for both importins were irreversible, as well as that of the complexes with any peptide. The apparent thermal denaturation midpoint was estimated from a two-state equilibrium equation as described [30].

### 2.5. CD

Far-ultraviolet (UV) CD spectra were collected on a Jasco J810 spectropolarimeter (Jasco, Tokyo, Japan) with a thermostated cell holder and interfaced with a Peltier unit at 298 K. The instrument was periodically calibrated with (+)-10-camphorsulphonic acid. A path length cell of 0.1 cm was used (Hellma, Kruibeke, Belgium). All spectra were corrected by subtracting the corresponding baseline. The concentration of each polypeptide was the same used in the fluorescence experiments. The buffer was the same used in the fluorescence experiments.

#### 2.5.1. Far-Ultraviolet (UV) Spectra

Isothermal wavelength spectra of each sample were acquired with six scans at a scan speed of 50 nm/min, with a response time of 2 s and a bandwidth of 1 nm. The samples were prepared the day before and left overnight at 278 K to allow for equilibration. Before starting the experiments, the samples were further left for 1 h at 298 K.

#### 2.5.2. Thermal Denaturations

The experiments were performed at 60 K/h and a response time of 8 s. Thermal scans were collected by following the changes in ellipticity at 222 nm from 298 to 343 K. The rest of the experimental set-up was the same as reported in the steady-state experiments. Thermal denaturations were not reversible for any of the samples, as shown by: (i) comparison of the spectra before and after the heating and (ii) changes in the voltage of the instrument detector [31]. The apparent thermal denaturation midpoint of the samples was estimated as described [30].

### 2.6. ITC

The experimental set-up and data processing of ITC experiments has been described previously [32]. Calorimetric titrations, performed in an Auto-iTC200 calorimeter (MicroCal, Malvern-Panalytical, Malvern, UK) consisted of series of 19 2-μL injections, with 150 s time spacing and a 750-rpm stirring speed. Impα3 or ΔImpα3 (at 10–20 μM) was loaded into the calorimetric cell and NLS-NUPR1 peptides in the syringe (150–300 μM); all solutions were prepared in buffer Tris 50 mM, pH 8. The temperature for all the experiments was 298 K. The experiments were analyzed by applying a model considering a single ligand binding site (1:1 stoichiometry) implemented in Origin 7.0 (OriginLab, Northampton, MA, USA). The binding affinity (association constant) and the binding enthalpy were estimated through a least-squares nonlinear regression data analysis, from which the Gibbs energy and the entropic contribution to the binding were calculated using well-known thermodynamic relationships. Since the binding stoichiometry is constrained by the model, the parameter n provides a fraction of the active or binding competent protein. Experiments for each peptide and importin species were performed, at least, in duplicates.

### 2.7. NMR

The NMR experiments were acquired at 283 K on a Bruker 500 MHz Advance III spectrometer (Bruker GmbH, Karlsruhe, Germany) equipped with a triple-resonance probe and z-pulse field gradients. Temperature of the probe was calibrated with methanol [33]. All experiments were carried out at pH 7.2, 50-mM deuterated Tris buffer (not corrected for isotope effects). The spectra were calibrated with TSP ((trimethylsilyl)-2,2,3,3-tetradeuteropropionic acid) by considering pH-dependent changes of its chemical shifts [33].

#### 2.7.1. 1D-^1^H-NMR (One-Dimensional Proton NMR) Spectra

In all cases, 128 scans were acquired with 16 K acquisition points and using concentrations of 1.0–1.2 mM. Homonuclear 1D-^1^H-NMR spectra were processed with Bruker TopSpin 3.1 (Bruker GmbH, Karlsruhe, Germany) after zero-filling and apodization with an exponential window.

#### 2.7.2. Translational NMR Diffusion Ordered Spectroscopy (DOSY)

Concentrations of peptides in all DOSY experiments were 120 μM, and 128 scans, where the gradient strength was varied, were acquired for each curve. Translational self-diffusion measurements were performed with the pulsed gradient spin-echo sequence in the presence of 100% D_2_O. Experimental details have been described elsewhere [30]. Briefly, the gradient strength was varied in sixteen linear steps between 2% and 95% of the total power of the gradient coil. The gradient strength was previously calibrated by using the value of the translational diffusion coefficient, *D*, for the residual proton water line in a sample containing 100% D_2_O in a 5-mm tube [34]. In our experiments for each peptide, the duration of the gradient was 2.25 ms, the time between the two pulse gradients in the pulse sequence was set to 200 ms, and the recovery delay between the bipolar gradients was set to 100 μs. The methyl groups with signals between 1.0 and 0.80 ppm were used for integration. Fitting of the exponential curves obtained from experimental data was carried out with KaleidaGraph (Synergy Software, Version 3.5), as described [30]. A final concentration of 1% of dioxane, which was assumed to have a hydrodynamic radius *R*_h_ = 2.12 Å [34], was added to the solutions of each of the peptides to have a comparison for estimating their sizes.

#### 2.7.3. 2D-^1^H-NMR Spectra

Two-dimensional spectra in each dimension were acquired in phase-sensitive mode by using the time-proportional phase incrementation technique (TPPI) and a spectral width of 7801.69 Hz [35]; the final concentration was the same used in the 1D-^1^H-NMR experiments. Standard total correlation spectroscopy (TOCSY) (with a mixing time of 80 ms) [36] and nuclear Overhauser effect spectroscopy (NOESY) experiments (with a mixing time of 250 ms) [37] were performed with a data matrix size of 4K × 512. The DIPSI (decoupling in the presence of scalar interactions) spin-lock sequence [38] was used in the TOCSY experiments with 1 s of relaxation time. Typically, 64 scans were acquired per increment in the first dimension, and the residual water signal was removed by using the WATERGATE sequence [39]. NOESY spectra were collected with 96 scans per increment in the first dimension, with the residual water signal removed again by the WATERGATE sequence and 1 s of relaxation time. Data were zero-filled and resolution-enhanced in each dimension, with a square sine-bell window function optimized in each spectrum, baseline-corrected, and processed with Bruker TopSpin 3.1. The ^1^H resonances were assigned by standard sequential assignment processes [40]. The chemical shift values of H_α_ protons in random-coil regions were obtained from tabulated data, corrected by neighboring residue effects [41,42] and taking into account the phosphorylation of Thr68 [43,44] for the corresponding peptide.

### 2.8. Molecular Docking

Molecular docking was performed using AutoDock Vina (Version 1.1.2) [45], largely following a protocol we have previously described for screening NUPR1 sequence fragments [24]. The structure of ΔImpα3 was modeled on the basis of the Protein Data Bank (PDB) entry 5 × 8N [46], which reports the X-ray structure of monomeric Impα1 bound to the NLS of the Epstein-Barr virus EBNA-LP protein. The search volume was centered on the macromolecule and had the size 50 Å × 90 Å × 90 Å, which was sufficient to carry out a blind search on the whole protein surface.

The peptides used in our experiments encompassed residues 53–74 of NUPR1, with a number of rotatable dihedral angles ranging from 85 to 91. Their conformational space was too large to be reasonably treated by molecular docking; therefore, we followed a two-fold approach [47] that consisted in reducing the number of degrees of freedom and using a longer search protocol. The number of rotatable dihedrals was halved by considering the reduced sequence that encompasses residues 63–71 of NUPR1, and therefore, it includes only the core region of the NLS. These shorter peptide sequences were capped with an acetyl and N-methyl group at the two main chain endings, to mimic the fact that they are internal portions of the sequence of the protein, as well as of their full-length parent peptides. An extensive search was performed with very high exhaustiveness, 16 times larger than the recommended default value [48].

## 3. Results

### 3.1. The Isolated wt NLS-NUPR1 and Its Mutants Were Monomeric and Disordered in Aqueous Solution

We first determined the conformational propensities of isolated peptides by using CD and NMR. We did not use fluorescence to characterize their conformational features, because the peptides only have a single tyrosine at their N terminus, whose maximum wavelength (~308 nm) does not change under different environments in solutions [49]. The CD spectra of isolated peptides did show an intense minimum at ~200 nm (Figure S1), indicating that they were mainly in a random-coil conformation. This was further confirmed by 1D-^1^H-NMR spectra, which showed, for all the peptides, a clustering of the signals of all the amide protons between 8.0 and 8.5 ppm (Figure S2) and grouping of the methyl protons between 0.8 and 1.0 ppm, which is a feature of disordered polypeptide chains [40].

The peptides were monomeric, as concluded from the values of *D* measured by the DOSYs and the calculation of the estimated *R*_h_ from a random-coil polypeptide according to an exponential law [29] (Table 1).

**Figure 1:**
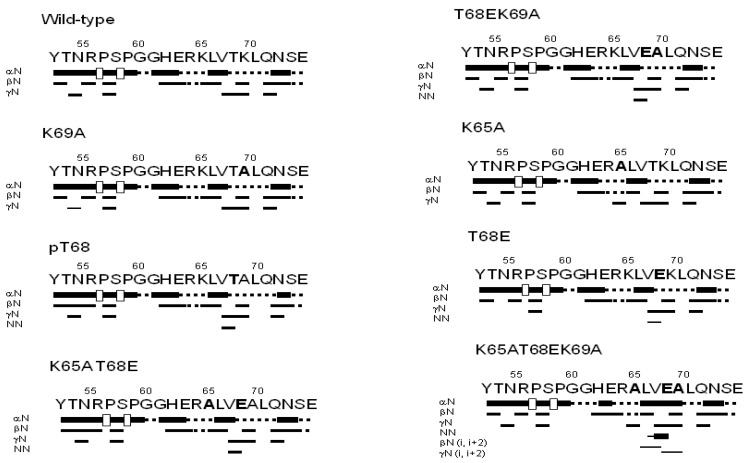
NMR structural characterization of the nuclear localization sequence (NLS) NUPR1 peptides. Nuclear Overhauser effects (NOEs) are classified into strong, medium, or weak, as represented by the height of the bar underneath the sequence; the signal intensity was judged by visual inspection from the nuclear Overhauser effect spectroscopy (NOESY) experiments. The symbols αN, βN, γN, and NN correspond to the sequential contacts (that is, for instance, the NN corresponds to the NN (*i*,*i* + 1) contacts). The corresponding H_α_ NOEs with the H_δ_ of the following proline residues are indicated by an open bar in the row corresponding to the αN contacts. The dotted lines indicate NOE contacts that could not be unambiguously assigned due to signal overlap. The numbering of the residues corresponds to that of the whole sequence of NUPR1.

To further confirm the disordered nature of the peptides, we also carried out homonuclear 2D-^1^H-NMR experiments (Tables S1–S8). For all peptides, NOEs between the H_α_ protons of Arg56 or Ser58 and the H_δ_ of the two following residues (Pro57 and Pro59, respectively) were always observed (Figure 1); these findings suggest that the Arg56-Pro57 and Ser58-Pro59 peptide bonds predominantly adopted a trans-conformation in all the peptides (other minor signals were not observed). Two lines of evidence confirmed the disordered nature of the peptides (further pinpointing the findings from far-UV CD (Figure S1) and the 1D-^1^H-NMR spectra (Figure S2)). First, the sequence-corrected conformational shifts (Δδ) of H_α_ protons [40,41,42,43,44] were within the commonly accepted range for random-coil peptides (Δδ ≤ 0.1 ppm) (Tables S1–S8). It is interesting to note at this stage that, in the phosphorylated Thr68 of the pT68 peptide, the signals from the H_β_ protons were downfield shifted when compared to those of the wt peptide (4.58 versus 4.15 ppm, respectively), as well as the chemical shift of the amide proton: 8.62 versus 8.33, respectively (Tables S1 and S3), as it has been reported to occur for phosphorylated threonines [43,44], thus confirming the phosphorylation of this particular threonine and not of the other one in the sequence, Thr54. Second, in any of the peptides, no long- or medium-range NOEs were generally detected but, rather, only strong sequential ones (αN(*i*,*i* + 1)) (Figure 1). Only in the pThr68 peptide and in the four phospho-mimics (T68E, K65AT68E, T68EK69A, and K65AT68EK69A peptides), we observed a weak NOE (NN(*i*,*i* + 1)) between the amide protons of Val67 and Thr68 (Figure S3). This NOE, although weak when compared with the intensity of sequential αN(*i*,*i* + 1) NOEs, is a fingerprint signature of turn-like conformations [40].

Although there are some isolated short peptides that are partially structured (such as the isolated Ribonuclease S peptide [33,40]), our findings by CD and NMR indicate that the isolated NLS-NUPR1 peptides were mainly disordered in aqueous solution when isolated.

### 3.2. The NLS-NUPR1 Peptides Bound to Both Impα3 and ΔImpα3

In the present work, we measured the affinity of intact NUPR1 for ΔImpα3, obtaining a value for the dissociation constant of 0.4 μM (Figure S4), and we have previously measured the affinity of intact NUPR1 for Impα3, and a value of 1.4 μM has been obtained (shown in Figure S4; for a comparison, [50]). Furthermore, we tried to dissect the affinity of the NLS region of NUPR1 for Impα3 by using a “divide and conquer” approach with the peptides comprising the region. The interaction between full-length NUPR1 and its mutants with Impα3 and ΔImpα3 was the focus of this study, but instead, we employed NLS peptides to elucidate the binding mechanism to Impα3. The reason behind such an approach relies in the fact that we have observed that, very often, mutations at any place of the polypeptide length of NUPR1 result in a poor expression of the corresponding mutant, and mutations in some positions lead to no expression at all [24].

**Figure 2:**
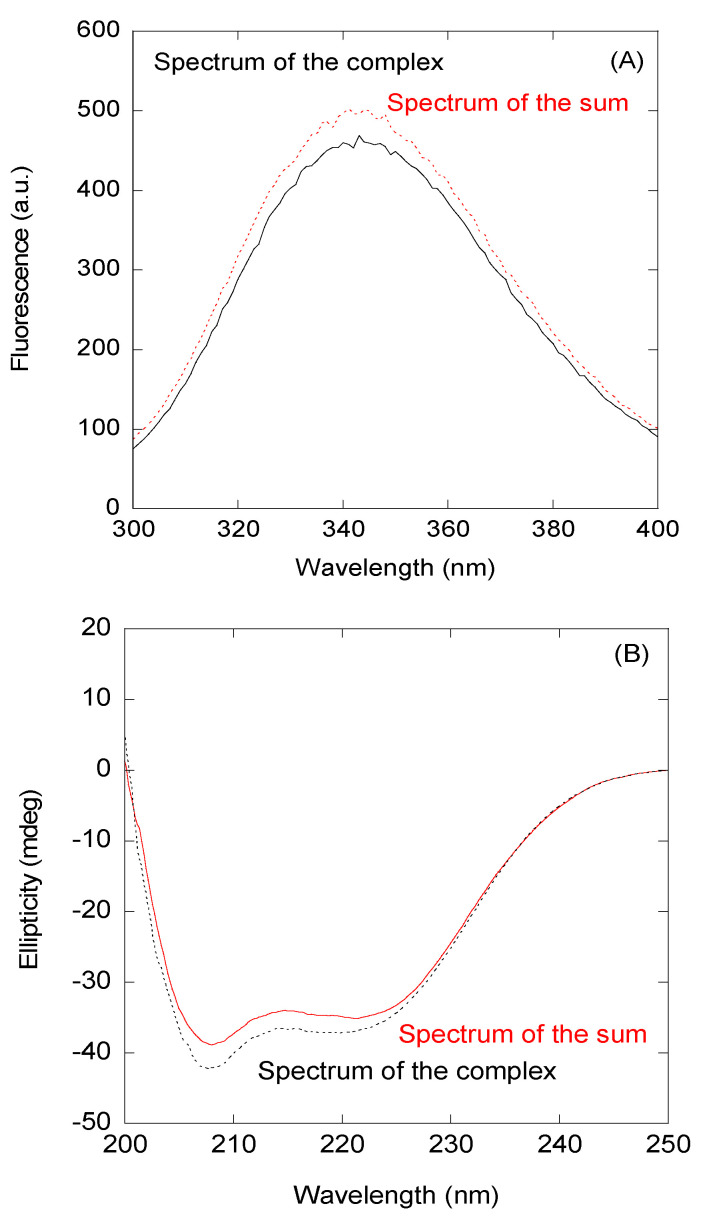
Binding of the wild-type (wt) peptide to importin α3 (Impα3) monitored by spectroscopic techniques: (**A**) Fluorescence spectrum obtained by excitation at 280 nm of the complex between Impα3 and the wt peptide and the addition spectrum obtained by the sum of the spectra of both isolated macromolecules. (**B**) Far-UV CD (ultraviolet circular dichroism) spectrum of the complex between the Impα3 and wt peptides and the additional spectrum obtained by the sum of the spectra of both isolated macromolecules.

First, we decided to investigate a possible interaction between the NLS NUPR1 peptides and Impα3 in vitro by using fluorescence and CD. As a representative example, we describe our findings for the wt peptide. We observed changes in the fluorescence spectrum of this peptide after excitation at 280 nm (whereas there were no changes at 295 nm); that is, the additional spectrum obtained from the spectra of isolated wt peptide and either Impα3 or ΔImpα3 was different to those of their respective complexes (Figure 2A). These results indicate that tyrosine residues of at least one of the biomolecules (peptides with either Impα3 or ΔImpα3) were mainly involved in the binding. The changes were small for Impα3, and there were no changes for ΔImpα3; furthermore, thermal denaturations followed by fluorescence did not show a variation in the apparent thermal denaturation midpoint for both Impα3 and ΔImpα3 (Figure S5). On the other hand, the comparison of the additional spectrum and that of the complex obtained by far-UV CD did show differences (both for Impα3 and ΔImpα3), indicating that there were changes in the secondary structure of at least one of the macromolecules upon binding (Figure 2B); however, there were no differences in the determined thermal denaturation midpoint for isolated Impα3 (or ΔImpα3) and that of the complex (Figure S5). It is important to note that the far-UV CD region is sensitive to elements of secondary structures (α-helix and β-sheet); however, local structural elements and nonregular structures might also be present, which could be masked by the presence of long disordered regions. The above results indicate that there was binding between the wt peptide and both importins, but the binding did not induce large changes in the structures of both macromolecules.

The situation was slightly different in the case of the Impα3 and ΔImpα3 complexes with the other mutant peptides. As an example, we described our results with the K65A peptide, and the findings for the other peptides were basically similar to those described here. Where the far-UV CD spectra of the addition and that of the complex with both importins also showed small differences (Figure S6A,B), the fluorescence spectra did not have modifications (either by excitation at 280 or 295 nm) (Figure S6C,D). In general, for the mutant peptides, the changes were smaller than for the wt peptides.

**Figure 3:**
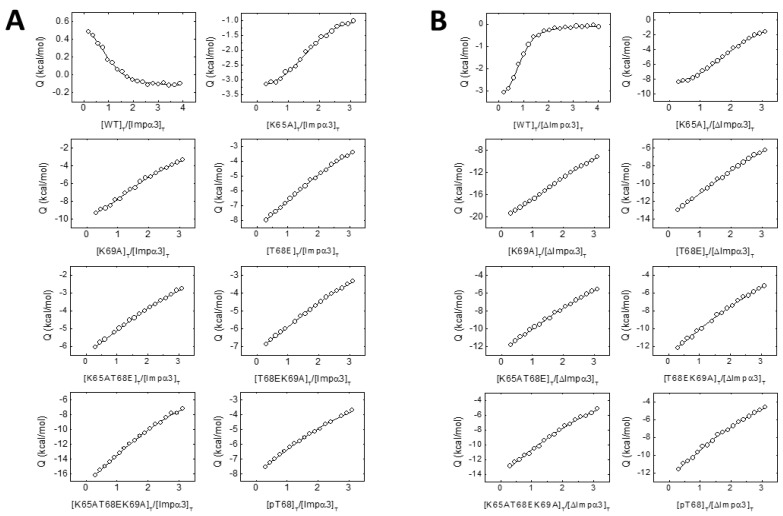
The interaction of the wt and mutant NLS NUPR1 peptides with both importins as measured by isothermal titration calorimetry (ITC). Interaction isotherms (ligand normalized heat effect per injection as a function of the ligand:protein molar ratio) with Impα3 (**A**) and ΔImpα3 (**B**) are shown. Binding parameters were estimated by a nonlinear least-squares regression data analysis of the interaction isotherms applying a single ligand binding site model, implemented in Origin 7.0.

The above experiments were sufficient to conclude that the NLS-NUPR1 peptides interacted with Impα3 or ΔImpα3, but we also carried out ITC experiments to measure the binding affinity. The results (Table 2 and Figure 3) indicate that: (i) the highest affinity towards either Impα3 or ΔImpα3 was that observed for the wt peptide, (ii) the affinity for most of the peptides was higher for binding to ΔImpα3 (the only exceptions were the T68EK69A and pT68 peptides), (iii) removal of Lys65 or Lys69 residues decreased the affinity (and the variations in affinity were higher for ΔImpα3 than for Impα3), and (iv) the phosphorylation or mutation to Glu (phospho-mimics) of Thr68 decreased the affinity by almost one order of magnitude when compared to the other mutations for both importin species. Therefore, the ITC findings mirrored the results obtained by fluorescence: there were lesser structural changes (as reported by fluorescence) in the binding of the peptide mutants than for the wt one, and the affinity of the former peptides for importins was lower (Table 2).

Taking together all these findings, we conclude that the isolated region of NUPR1 comprising its NLS was capable of binding to Impα3 and that this binding was strongly modulated by the phosphorylation state of Thr68 and the charges at positions Lys65 and Lys69.

### 3.3. Binding Regions in the Docking of NUPR1 Peptides to Importins

Since we have shown that there was binding between the peptides and both importins, and we have identified the most important residues for attaining such binding, we performed molecular docking to determine details on the location and binding energy of the NUPR1 peptides on the surface of Impα3. When applied to our case, the docking techniques possess three caveats that are worth mentioning explicitly. First, even in the case of our relatively short peptides, the number of degrees of freedom to be considered is too large to be computationally tractable. This number was halved by considering reduced sequences (nine amino acids, corresponding to residues 63–71 of NUPR1), which included all the mutation sites plus at least two more residues at each end. Second, it is impossible with this technique to discriminate differences in the binding between Impα3 and ΔImpα3, and therefore, only the latter protein structure was considered. Third, molecular docking does not take into account the dynamics of a protein-ligand complex, which could also contribute to the binding. Keeping in mind these limitations, the protein surface was blindly explored by considering a volume that included the whole structure and using a high exhaustiveness of search that is equivalent to running multiple (>10) distinct simulations.

**Figure 4:**
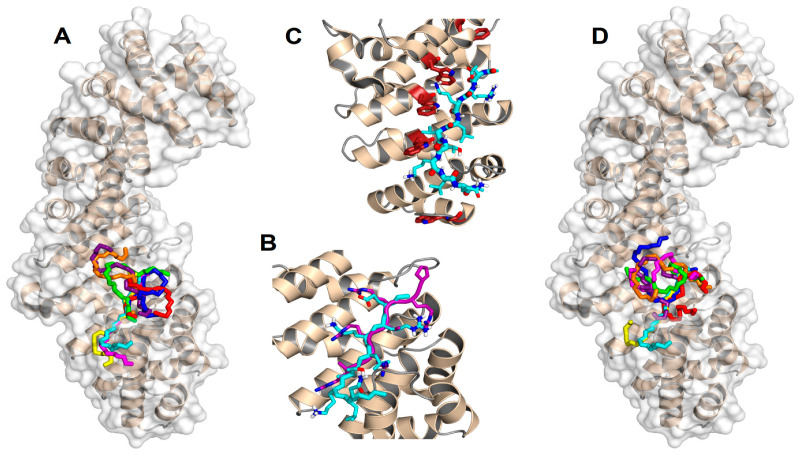
Predicted docking poses for the NLS of NUPR1 on importin. (**A**) Backbone (–N–C^α^–C– atoms) representation of the best eight docking poses on ΔImportin for the wt sequence ERKLVTKLQ (the N terminus is on the top), which constitutes the core region for the NLS of NUPR1. (**B**) Most favorable binding pose for the same sequence (cyan), compared to the crystallographic conformation [46] of the NLS of the Epstein-Barr virus EBNA-LP protein (purple). For clarity, atoms are shown in standard colors only in the side chains of the two peptides, and the main-chain O and H atoms are omitted; apolar H atoms are not present. (**C**) Trp residues (brown) in the major NLS-binding site of importin play a key role in the binding of the most favorable conformation of the NLS of wild-type NUPR1. The view is slightly rotated with respect to previous representations to evidence the tryptophan side chains. (**D**) Most favorable docking poses for the eight peptide sequences: wild type (cyan), K65A (magenta), K69A (yellow), T68E (blue), K65AT68E (red), T68EK69A (green), K65AT68EK69A (orange), and pT68 (violet). PyMol was used for all displays.

Figure 4 summarizes the predictions obtained in our docking calculations. In particular, Figure 4A illustrates the energetically most favorable poses obtained for the wild-type (capped) sequence ERKLVTKLQ mapped on the surface of importin. The best eight poses are reported for clarity and to obtain a more direct comparison with the cluster of the single best pose for each of the eight different peptides (see below, Figure 4D). The results clearly show that the most favorable binding modes cluster into a single location that consists of the major NLS-binding site, located on ARM repeats 2–4. As shown in Figure 4B, the best structure found for our peptide sequence overlaps quite remarkably with that of the NLS of the Epstein-Barr virus EBNA-LP protein, whose structure has been previously determined in crystallography [46]. A number of different amino acids participate in the binding, including some key tryptophan residues (see the details in Figure 4C) that are known to play an important role in the formation of the importin-cargo complex. The binding energy in the docking for the most favorable conformation was −7.2 kcal/mol, indicating a moderate affinity in the low micromolar range. Compared to the experimental values found for the whole wild-type sequence YTNRPSPGGHERALVTKLQNSE (−7.87 and −8.22 kcal/mol for Impα3 and ΔImpα3, respectively; see Table 2), this finding indicates that the reduced docked sequence provides the major contribution to the binding-free energy of the full-length peptide.

Figure 4D shows the best docking poses obtained for the seven mutant sequences compared to the wt one, which is also reported. Again, in this case, all the most favorable binding modes (and, more generally, even the first ten docking poses for each peptide species) clustered in the same location correspond to the major NLS-binding site. This observation suggests that the mutations do not modify essentially the binding location of the peptides but only their affinity towards importin. The calculated binding energies ranged from −5.6 to −6.6 kcal/mol, indicating that any of the explored mutations reduced the binding affinity with respect to the wt sequence, in agreement with our experimental results (Table 2). We observed a poor correlation between the computational and experimental rankings of the mutated peptides in terms of affinity towards the protein, although this could reasonably be explained, because the experimental binding energies are, in most cases, very close to each other (Table 2). This finding did not let us push too far the interpretation of our results in terms of the molecular details that assist the binding. Nevertheless, the contribution of the protein tryptophan residues to the binding still seemed to be, in all cases, an important determinant (even though we did not observe changes in the fluorescence spectra (either by excitation at 280 or 295 nm) when binding for some of the mutant peptide sequences was explored, Figure S6).

To sum up, a number of important conclusions can be drawn from the docking results reported: (i) all the sequences investigated interacted with the same region of importin; (ii) this region matched unambiguously with the major NLS-binding site of the protein; (iii) the ligand with the highest binding affinity corresponded to the wt sequence of NUPR1 (in agreement with the experimental results from ITC; Table 2); (iv) the major contribution to the binding energy of the parent peptides (i.e., those used in this work) was due to such a restricted sequence portion, which includes only nine residues (and this region includes Lys65, Thr68, and Lys69); (v) this essential sequence fragment corresponded to the core region of the predicted NLS of NUPR1; (vi) the binding region roughly mapped around Thr68 (where the residue name and number refers to wild-type, intact NUPR1 numbering), which therefore appears to be a key amino acid; and (vii) the most favorable predicted structure for the NLS region of wild-type NUPR1 essentially overlapped with the conformation of the NLS of a different protein (the Epstein-Barr virus EBNA-LP protein) determined in crystallography.

## 4. Discussion

### 4.1. Molecular Mechanisms for Impα3 Recognition of NUPR1: The Influence of Lys65 and Lys69

In this work, we tried first to find out whether the theoretically predicted NLS region of NUPR1 was capable of binding in isolation to Impα3. Second, we tried to elucidate, for the first time, the molecular bases behind the binding of an NLS region of an IDP to an importin. Our results indicate that the isolated, wild-type NLS region of NUPR1 interacted with the intact Impα3 and ΔImpα3, with an affinity similar to that for intact NUPR1 (1.4 μM, Figure S4), and within the same range measured for the affinities to natural partners of NUPR1 [22,24,51] and synthetic molecules [23,52]. Furthermore, our results also address the molecular importance of IBB in the binding of cargos to importins.

As it happens for the intact NUPR1 (whose dissociation constants are 1.4 μM for Impα3 and 0.44 μM for ΔImpα3 (Figure S4)), the wt peptide bound to ΔImpα3 with a two-fold larger affinity (0.95 μM) than that for Impα3 (Table 2) (1.7 μM). These findings allow us to draw several conclusions. First, the presence of the IBB region (which contains a large quantity of lysine amino acids) exerts an autoinhibitory effect, and the domain hampers the entrance of the NLS peptide into the major NLS-binding region of Impα3, as it has been suggested in other studies with well-folded proteins [9]. However, this is the first time such a hypothesis is tested in an IDP. Modulation of the assembly complex formation between importins and their cargos has been attributed to the IBB domain [4]; this domain has been found to be involved even in the formation of a homodimeric species between importins [53], with a reduced ability to bind cargos. Second, although the affinities of the wt peptide for both importins were smaller than those for intact NUPR1, many of the interactions implicated in the binding to importin could be ascribed to a region comprised within the wt peptide, as concluded from the similarities among the dissociation constants (0.44 (intact NUPR1) and 0.95 μM (wt peptide) for ΔImpα3 and 1.4 (intact NUPR1) and 1.7 μM (wt peptide) for Impα3). Third, given the similarities among the affinity constants for Impα3 of the wt peptide and NUPR1, the peptide could be used as a lead compound to design an inhibitor of its nuclear translocation.

We have previously shown in vivo that a mutant of NUPR1 at positions Lys65, Lys69, Lys76, and Lys77 is present through the whole cell, whereas the wild-type NUPR1 species is localized exclusively into the nucleus [25]. In this work, we have found that the mutation Lys65Ala decreased two-fold the affinity for Impα3, and the mutation Lys69Ala decreased six-fold the affinity. Thus, the decrease in the affinity was larger with the removal of Lys69, probably indicating that this residue makes more contacts with importin, as pinpointed by our docking models. In fact, we observed in the simulation that both lysine residues were involved in hydrophobic and polar contacts (the latter with their NH_3_^+^ moieties), with residues of importin α. The removal of the long side chains would disfavor those contacts, thus decreasing the affinity (Table 2). The importance of lysines is key in determining the binding to importins of other well-folded proteins through their disordered NLS regions, as shown by several structural studies [3,8,54,55]. It could be thought that our study does not provide new mechanistic insight into the function of importins, because the results obtained with an IDP pinpoint, for the first time, the importance of positive charges (as it happens in folded proteins) in the binding of their cargos; however, to the best of our knowledge, this is the first reported case where the importance of such residues is addressed in vitro for the NLS of an IDP, and our results acquire more relevance considering recent findings, where it has been suggested that IDPs do not require the presence of importins to be translocated into the nucleus, although demonstrated mostly for acidic proteins [56]. Then, our results indicated that IDPs require the help of importins to be translocated into the nucleus, and it seems that the rules governing such processes are similar to those observed in well-folded proteins.

The same decrease in affinity was observed for the K65A and K69A peptides towards ΔImpα3, but, compared to the wt peptide, the variation was larger than that observed for both mutants with Impα3 (Table 2). Furthermore, as it happens with the intact importin, the decrease in affinity was larger for the K69A peptide. These findings indicate that, although the IBB region maintains its independence within the whole Impα3 in terms of conformation, its removal may either alter the structure of some regions of the ARM repeats involved in the major NLS-binding site (which relies on hydrophobic contacts to anchor the cargo, therefore altering its docking) or, alternatively, IBB removal may change the whole protein dynamics and its stability.

### 4.2. Molecular Mechanisms for Impα3 Recognition of NUPR1: The Influence of Thr68 and Its Phosphorylation-Triggered Conformational Switch

Apart from the importance of the two lysines of NUPR1 in the binding to importins, we also wanted to address the importance of Thr68. It is well-established that Thr68 is a key residue in the binding of NUPR1 to any partner, either natural or synthetic [22,23,24,57]; in fact, together with Ala33, it constitutes one of the two hotspot regions of NUPR1. We decided to address such a question by following two approaches: (i) we mutated Thr68 to Glu to have the phospho-mimics, and (ii) we synthesized a peptide with the phosphorylated Thr (pT68 peptide).

Among all the mutants explored, the peptide with the smallest affinity for Impα3 or ΔImpα3 (~30 μM for both species) was the pThr68 peptide (Table 2). Phosphorylation affects the binding probably by inhibiting long-range electrostatic contacts with both importins. Where the affinity of the wt peptide for ΔImpα3 was larger, the changes due to the addition of the phosphate group in the pThr68 peptide were even larger, further pinpointing subtle structural changes in the major NLS-binding region upon removal of the IBB. Around a third of the eukaryotic proteins can be phosphorylated, and the majority of those phosphorylation sites belong to intrinsically disordered regions because of their accessibility to kinases [14]. Phosphorylation is a key regulatory mechanism in translation, transcription, and other processes.

The phospho-mimetic peptide of NLS-NUPR1, the T68E peptide, also showed a smaller affinity for both importins than the wt one (22 μM for Impα3 and 12 μM for ΔImpα3, Table 2), but the decrease was not as large as that in the pT68 peptide (27 μM for Impα3 and 29 μM for ΔImpα3, Table 2), indicating that the phospho-mimics did not cause the same effect as phosphorylation. Phosphorylation at Thr68 replaces the neutral OH (hydroxyl) group with a tetrahedral PO_4_^2−^ (phosphoryl group) with two negative charges, which modifies the electrostatic, chemical, and steric properties of the threonine environment. The double-negative charge of the PO_4_^2−^ and its large surrounding hydration shell make the situation chemically different from the Glu phospho-mimic, which has a smaller hydration shell and a single negative charge. Differences among the affinities of phospho-mimics and phosphorylated threonines for a well-folded protein have been also observed in the affinities measured in other protein systems [58], as well as in other IDPs [59].

The values of the affinity constants of the K65AT68E and T68EK69A peptides were similar to that of the T68E peptide (Table 2). This finding indicates that: (i) the effect of Thr68Glu in the binding to importins surpassed those caused by substitutions of the single lysines (and then, Thr68 must have a greater importance in the interaction), and (ii) the effect of removing a lysine when the threonine is phospho-mimicked is not additive for the double mutants, probably because the remnant lysine establishes electrostatic interactions with the glutamic residue. However, the accumulation of the three mutations (in the K65AT68EK69A peptide) led to a large decrease of the affinity constant (Table 2), further highlighting the influence of electrostatic effects between the lysines and the phospho-mimics in the bindings with the two importins. Other studies of phosphorylation of threonines in IDPs indicate that the proximity of arginines can stabilize the charge of the phosphoryl moiety and the stabilization of turn-like structures [60]. We suggest that, in the case of NUPR1, lysines, instead of arginines, would play the role of stabilizing the conformation.

Interestingly enough, the peptides containing the phospho-mimic mutation (T68E) or the phosphorylated Thr68 (pT68 peptide) did show an NN(*i*,*i* + 1) NOE (Figure 1) between Val67 and phosphorylated Thr68 (or Glu68). We did not observe such a NOE in the intact NUPR1 when we assigned it [22]. It could be thought that the absence of such a NOE in the wt peptide may be due to the fact that the chemical shifts of the amide protons of those residues (Val67 and Thr68) were similar (Table S1), and then, the NOE could not be observed because of its proximity to the spectrum diagonal. However, the chemical shifts of amides of both residues in the K65A peptide were different enough (8.25 and 8.35 ppm for Val67 and Thr68, respectively; Table S6) to allow for its detection, and nevertheless, we did not observe any NOE (Figure S3). Thus, the presence of such a NOE, although it is weak in intensity, indicates that, upon phosphorylation, the two residues populated a turn-like conformation [40]; the presence of this turn is further supported by the observation of βN(*i*,*i* + 2) and γN(*i*,*i* + 2) NOEs and an additional NN(*i*,*i* + 1) contact for the K65AT68EK69A peptide involving residues Leu66–Thr68 (Figure 1), due to the large, intrinsic propensity of alanine to populate helix-like conformations [61,62]. As the affinity of the peptides for both importins decreased when Thr68 was phosphorylated or was phospho-mimicked (Table 2), we can conclude that the decrease in the affinity of peptides upon phosphorylation was structurally related to a conformational switch around Thr68, as a consequence of the introduced negative charge, shifting the population at equilibrium from a random-coil conformation to a turn-like one. The decrease in affinity for both importins may be related to the reduction in entropy of the polypeptide chain upon acquisition of the turn-like conformation and a concomitant conformational energetic penalty for the binding. Interestingly enough, two decades ago, we showed by using FTIR (Fourier transform infra-red spectroscopy) and CD that the unspecific phosphorylation of the serines and threonines in NUPR1 led to a higher population of α-helix- and/or turn-like conformations in the intact protein [21]; at the moment, however, we do not have any evidence for the biological importance of the particular phosphorylation of Thr68 in vivo. Nonetheless, we have recently shown that the mutation of Thr68 to Gln hampers the formation of several complexes of NUPR1 with other proteins involved in SUMOylation processes [50]. Our previous result is confirmed in this work by our new findings obtained with Thr68. Phosphorylation, as well as other post-translational modifications, can affect protein conformations: (i) on a local scale—for instance by affecting the population of *cis* proline isomers [63], (ii) determining a change of entropy of the conformational ensemble [64], (iii) modulating the binding to other macromolecules and triggering phase separation [65], (iv) in an allosteric manner, by affecting distant residues from the phosphorylation site [66], and, (iv) causing a conformational change [67,68]. Conformational switching affecting a threonine in several IDPs has been described [59,69,70]. For instance, the phosphorylation of Thr51 in the IDP prostate-associated gene protein increases the population of transient turn-like populations [70]; the difference with our results is that the turn-like structures in NUPR1 were stabilized in a much shorter polypeptide region, although we cannot rule out that phosphorylation at other sites of NUPR1 could help in stabilizing this conformation. On the other hand, the p27 protein, which modulates the mammalian cell cycle by the inhibition of cyclin-dependent kinases, contains some disordered regions, and the phosphorylation of residue Thr157 in breast cancer cells prevents its interaction with the nuclear import machinery, leading to the accumulation of this protein in the cytoplasm, whereas it is normally found in the nucleus [69]; however, no indication on the particular structure acquired upon phosphorylation at Thr157 has been provided. Finally, it is important to note that recent theoretical molecular dynamic simulations have shown that the binding of importin α to heterochromatin protein 1 α is modulated by phosphorylation at residues in its importin-binding region [71].

Thr68 is, together with the polypeptide patch around Ala33, the hotspot region of NUPR1, involved in binding to its natural partners [21,22,24,51] and to other synthetic molecules and macromolecules [23,52,57]. We have previously observed that the mutation of Thr68 to glutamine hampers the binding to those other molecules [24,51]. Such a mutation will probably cause a shift of the ensemble population from a random-coil towards turn-like conformations, and it is the adoption of such a local fold that hampers bindings to those other natural partners or synthetic molecules. Moreover, as the affinity of NUPR1 to its partners is basically the same in all cases described to date [21,22,24,51,52,57], its binding features can also be modulated by phosphorylation at Thr68 at least partially, since the region around Ala33 is also involved in the binding. In addition, since this threonine is also associated with the binding of drugs strongly effective against pancreatic cancer in mice [23,32], we hypothesize that the molecular effects of such drugs could be the induction of a stable fold (turn-like) by this polypeptide region, besides competitive steric hindrance, preventing binding to other natural partners of NUPR1, and hampering the protein cascades where it is involved.

## 5. Conclusions

We have described the interaction between the NLS region of NUPR1, a nuclear intrinsically disordered protein involved in cancer, and Impα3 by using a series of peptides comprising that polypeptide patch. Binding to Impα3 is modulated by the charges of Lys64 and Lys69 but, most importantly, by phosphorylation at Thr68, which constitutes an entropy-driven conformational switch, shifting the population of the dynamic ensemble towards a turn-like conformation. As Thr68 is also a hotspot for NUPR1 interactions, these results open the venue to modulating the binding to its partners by targeting this residue. Furthermore, it also suggests a possible mechanism for the action of drugs targeting NUPR1, which also bind through Thr68.

## Figures and Tables

**Table 1:** Hydrodynamic properties of the nuclear localization sequence (NLS) NUPR1 peptides.

Peptide ^a^	*D* (cm^2^ s^−1^) × 10^6^ (*R*_h_, Å) ^b^	*R*_h_, Å ^c^
YT^54^NRPSPGGHERKLVTKLQNSE (wt)	1.85 ± 0.04 (11 ± 1)	13 ± 3
YTNRPSPGGHER**A**LVTKLQNSE (K65A)	1.94 ± 0.08 (11 ± 1)	13 ± 3
YTNRPSPGGHERKLVT**A**LQNSE (K69A)	1.79 ± 0.06 (12 ± 2)	13 ± 3
YTNRPSPGGHERKLV**E**KLQNSE (T68E)	2.17 ± 0.06 (10 ± 1)	13 ± 3
YTNRPSPGGHER**A**LV**E**KLQNSE (K65AT68E)	1.76 ± 0.06 (12 ± 1)	13 ± 3
YTNRPSPGGHERKLV**EA**LQNSE (T68EK69A)	1.87 ± 0.08 (11 ± 1)	13 ± 3
YTNRPSPGGHER**A**LV**EA**LQNSE (K65AT68EK69A)	2.4 ± 0.2 (9 ± 2)	13 ± 3
YTNRPSPGGHERKLV**pT**KLQNSE (pT68)	1.89 ± 0.08 (11 ± 1)	13 ± 3

^a^ Mutations with respect to the wild-type sequence are indicated in bold. The last peptide has a phospho-threonine at position 68 (indicated with a “pT”). ^b^ The *R*_h_ was determined from the translational diffusion coefficient of dioxane (*R*_h_ = 2.12 Å) added to each sample. ^c^ Calculated from the scale law: *R*_h_ = (0.027 ± 0.01) MW^(0.50 ± 0.01)^ [29], where MW is the molecular weight of the peptide. *D*: translational diffusion coefficient.

**Table 2:** Thermodynamic parameters at 298 K in the binding reaction of NLS NUPR1 peptides to the two importin species ^a^.

	Impα3				ΔImpα3			
Peptide	*K*_d_ (μM)	Δ*H* (kcal/mol)	−*T*Δ*S* (kcal/mol)	*n*	*K*_d_ (μM)	Δ*H* (kcal/mol)	−*T*Δ*S* (kcal/mol)	*n*
wt	1.7	0.8	−8.7	0.9	0.95	−3.7	−4.5	1.0
K65A	3.9	−2.8	−4.6	1.4	2.7	−10.2	2.6	1.4
K69A	11	−10.8	4.0	1.3	7.6	−21.3	14.3	1.4
T68E	22	−11.1	4.7	(1)	12	−17.5	10.8	(1)
K65AT68E	21	−7.8	1.4	(1)	14	−17.9	11.3	(1)
T68EK69A	17	−7.5	1.0	(1)	17	−21.2	14.7	(1)
K65AT68EK69A	27	−16.3	9.1	(1)	24	−28.5	22.2	(1)
pT68	27	−14.8	3.6	(1)	29	−28.2	22.0	(1)

^a^ Relative error in *K*_d_ (dissociation constant) is 30%, absolute errors in Δ*H* (enthalpy) and −*T*Δ*S* (entropy) are 0.5 and 0.7 kcal/mol, respectively, and absolute error in n (the stoichiometry) is 0.2. The parenthesis in n values indicate that this parameter had to be fixed in order to get convergence in the fit due to low affinity.

## Supplementary Materials

The following are available online at https://www.mdpi.com/2218-273X/10/9/1313/s1, Figure S1: Structural features of NLS-NUPR1 peptides as monitored by far-UV CD, Figure S2: Structural features of NLS-NUPR1 peptides as monitored by 1D-^1^H-NMR, Figure S3: The amide region of 2D-^1^H-NOESY spectra of NLS-NUPR1 peptides, Figure S4: Interaction between (left) Impα3 and (right) ΔImp α 3 with full-length NUPR1 as observed by ITC, Figure S5: Thermal denaturation of the complexes followed by spectroscopic techniques, Figure S6: Interaction between Imp α3 and ΔImp α3 with K65A peptide measured by different spectroscopic techniques, Table S1: Chemical shifts (δ, ppm from TSP) of wt peptide in aqueous solution (pH 7.2, 283K), Table S2: Chemical shifts (δ, ppm from TSP) of K69A peptide in aqueous solution (pH 7.2, 283 K), Table S3: Chemical shifts (δ, ppm from TSP) of pT68 peptide in aqueous solution (pH 7.2, 283 K), Table S4: Chemical shifts (δ, ppm from TSP) of T68EK69A peptide in aqueous solution (pH 7.2, 283 K), Table S5: Chemical shifts (δ, ppm from TSP) of K65AT68E peptide in aqueous solution (pH 7.2, 283 K), Table S6: Chemical shifts (δ, ppm from TSP) of K65A peptide in aqueous solution (pH 7.2, 283 K), Table S7: Chemical shifts (δ, ppm from TSP) of T68E peptide in aqueous solution (pH 7.2, 283 K), Table S8: Chemical shifts (δ, ppm from TSP) of K65AT68EK69A peptide in aqueous solution (pH 7.2, 283 K).
