# Supplementary material for: A Phosphorylation-Induced Switch in the Nuclear Localization Sequence of the Intrinsically Disordered NUPR1 Hampers Binding to Importin
Source: Biomolecules. 2020 Sep 11;10(9):1313. doi: 10.3390/biom10091313 (PMC7565984; doi:10.3390/biom10091313)
Supplement: Supplementary file 1 [file biomolecules-10-01313-s001.pdf]

Supplementary Information for the manuscript

A phosphorylation-induced switch in the nuclear localization sequence of the intrinsically disordered NUPR1 hampers binding to importin

José L. Neira, Bruno Rizzuti, Ana Jiménez-Alesanco, Martina Palomino-Schätzlein, Olga Abián, Adrián Velázquez-Campoy and Juan L. Iovanna

SUPPLEMENTARY FIGURE LEGENDS

FIGURE S1: **Structural features of NLS-NUPR1 peptides as monitored by far-UV CD**: The spectra of selected NLS-NUPR1 peptides (indicated by different colours). Experiments were performed at pH 7.2 (50 mM Tris) and 283 K.

FIGURE S2: **Structural features of NLS-NUPR1 peptides as monitored by 1D-^{1}H-NMR**: The amide and aromatic region of the 1D-^{1}H-NMR spectra of selected NLS-NUPR1 peptides (indicated by different colours and some of them different to those whose CD spectra are shown in the previous figure). Experiments were performed at pH 7.2 (50 mM Tris) and 283 K. The broad signal appearing around 8.0 ppm in the spectra of some of the peptides is due to an impurity from the organic peptide synthesis, but it does not result in any cross-peak in the amide-amide region of the 2D-^{1}H-NOESY spectra (see next figure) or in another region of the 2D spectra acquired (NOESY or TOCSY).

FIGURE S3: **The amide region of 2D-^{1}H-NOESY spectra of NLS-NUPR1 peptides**: The presence of the NN(i,i+1) NOE is indicated with asterisks (*), because NOE positions change with the chemical shifts in each peptide. The colours of the different peptides are shown in the figure and they are the same as in Figure S2. All the spectra have been drawn with the same lowest contour level. Experiments were acquired at pH 7.2 (50 mM Tris) and 283 K.

FIGURE S4: **Interaction between (left) Impα3 and (right) ΔImpα3 with full-length NUPR1 as observed by ITC**: ITC raw data or thermogram (top: thermal power as a function of time) and titration curve or binding isotherm (bottom: ligand-normalized injection heats as a function of the reactants molar ratio) evidencing the interaction between Impα3 and ΔImpα3 with NUPR1. The dissociation constants for the interactions were estimated by non-linear least squares regression analysis considering a single ligand binding site model; analysis was performed by using Origin 7.0.

FIGURE S5: **Thermal denaturation of the complexes followed by spectroscopic techniques.** (A) Thermal denaturation followed by the changes in emission fluorescence after excitation at 280 nm of isolated Impα3 and that of its complex with the wt peptide. (B) Thermal denaturation followed by the changes in the raw ellipticity at 222 nm of isolated Impα3 and that of its complex with the wt peptide.

FIGURE S6: **Interaction between Impα3 and ΔImpα3 with K65A peptide measured by different spectroscopic techniques**: (A) Far-UV CD spectrum of the complex between Impα3 and K65A peptide and the addition spectrum obtained by the sum of the spectra of the two isolated macromolecules. (B) Far-UV CD spectrum of the complex between ΔImpα3 and K65A peptide and the addition spectrum obtained by the sum of the spectra of both isolated macromolecules. (C) Fluorescence spectrum obtained by excitation at 280 nm of the complex between Impα3 and K65A peptide and the addition spectrum obtained by the sum of the spectra of the two isolated macromolecules. (D) Fluorescence spectrum obtained by excitation at 280 nm of the complex between ΔImpα3 and K65A peptide and the addition spectrum obtained by the sum of the spectra of the two isolated macromolecules.

Fig. S1 (Neira et al.)

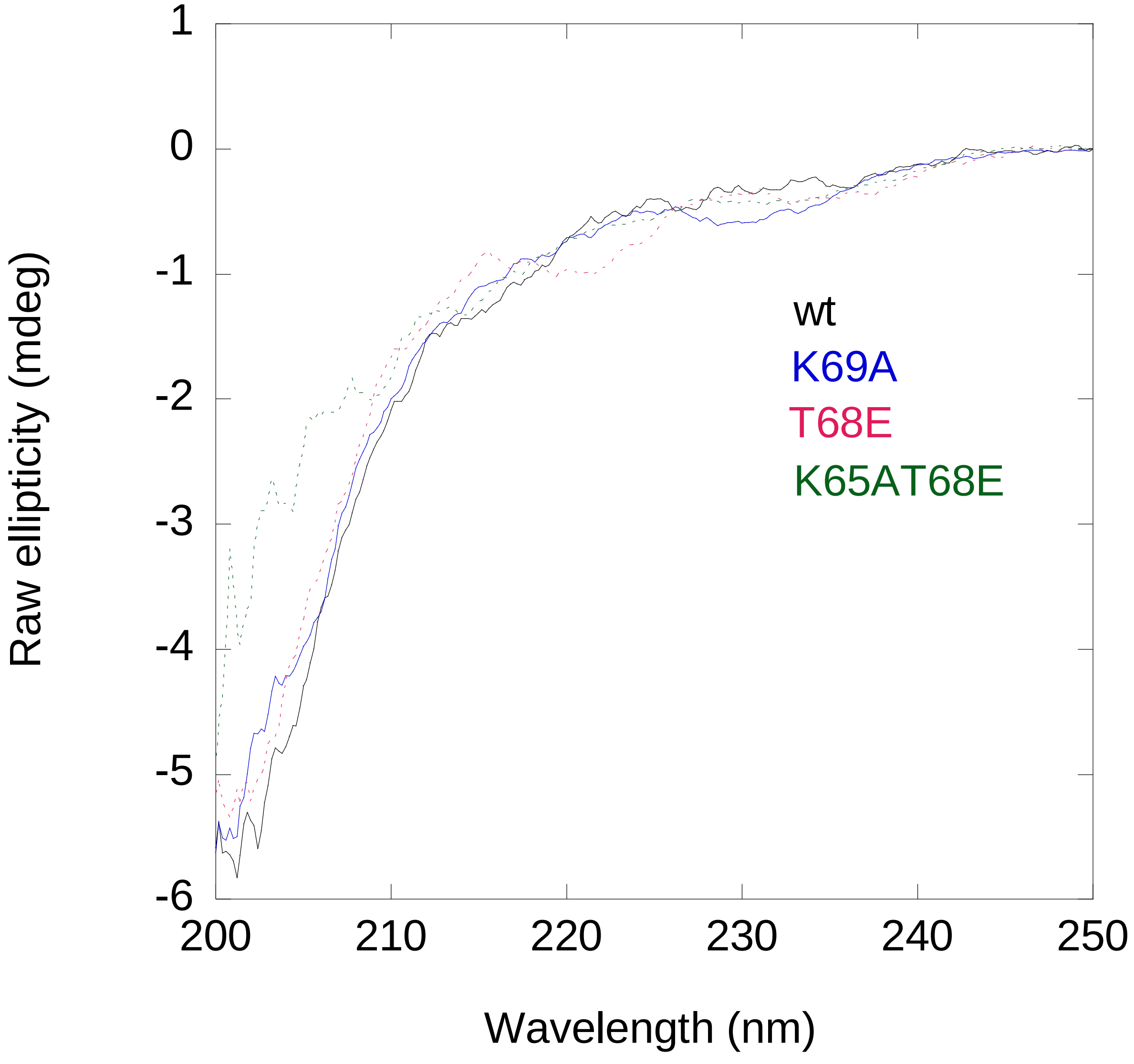

Fig. S2 (Neira et al.)

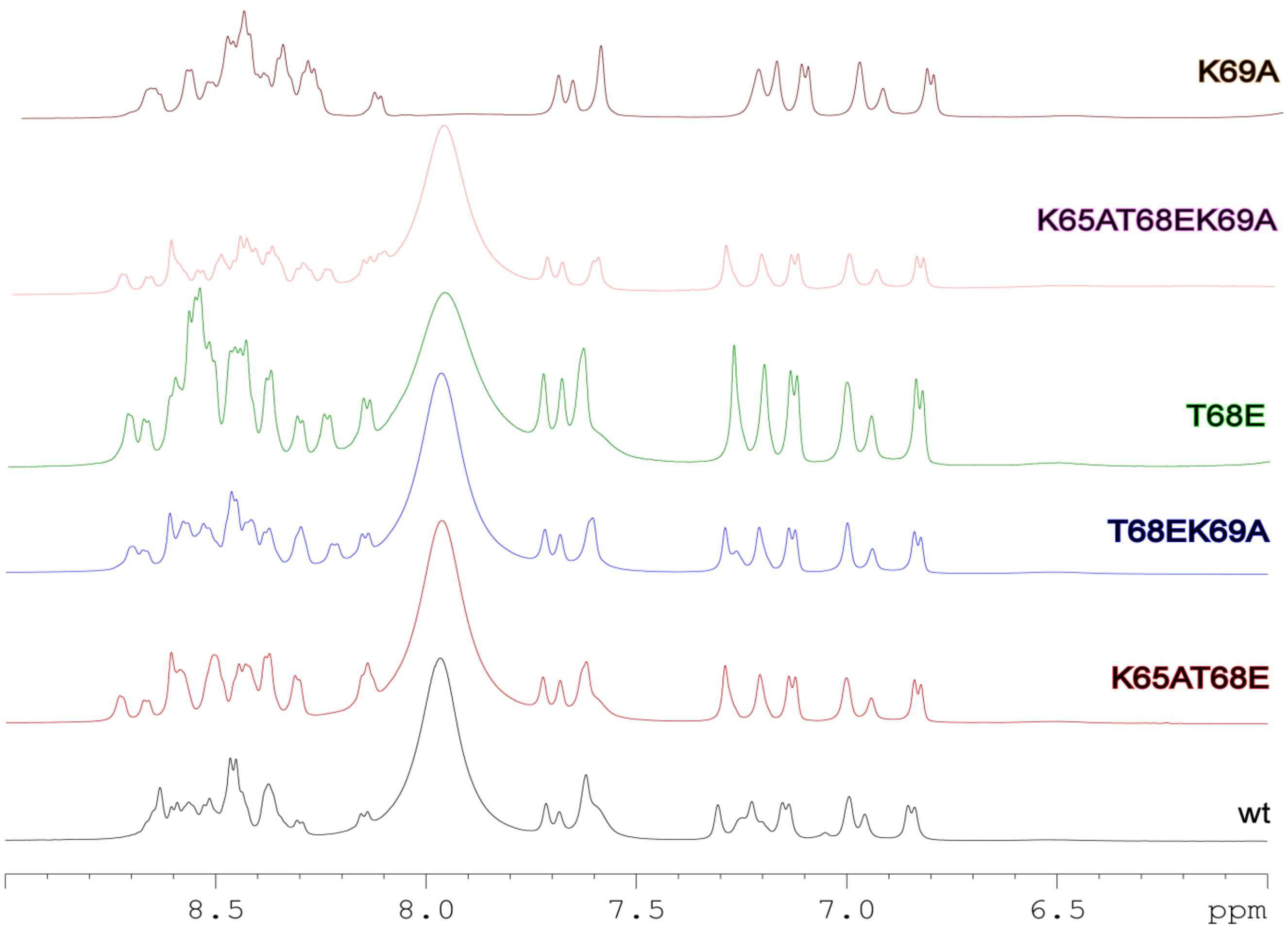

Fig. S3 (Neira et al.)

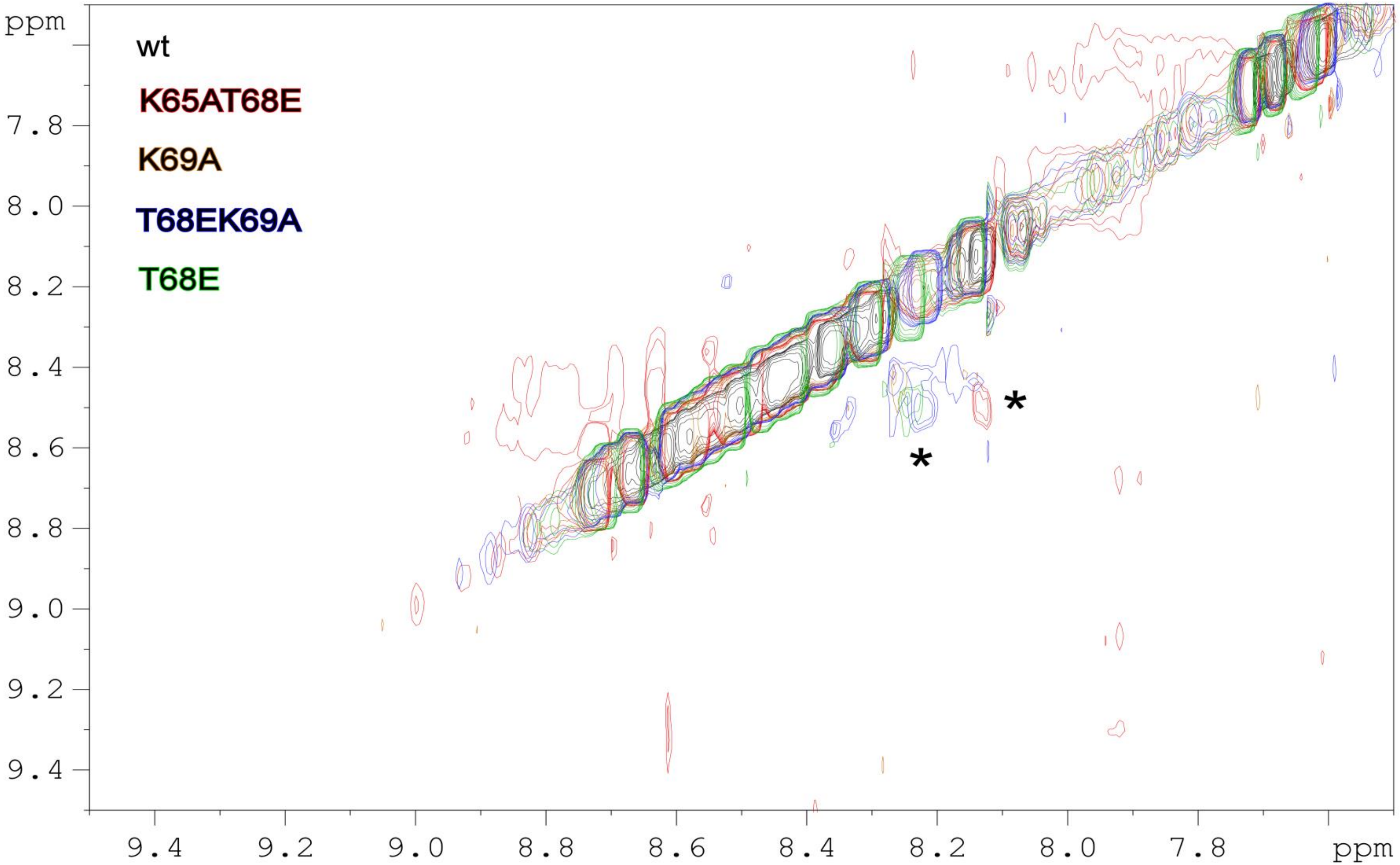

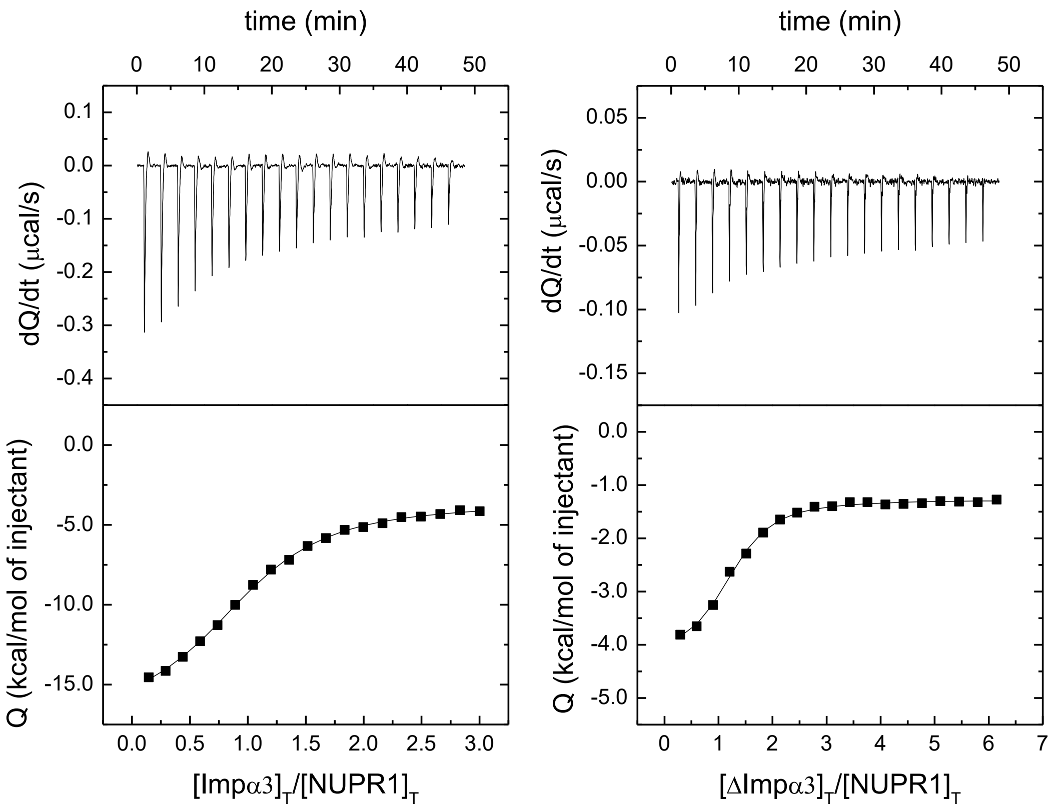

Fig. S4 (Neira et al.)

Fig. S5 (Neira et al.)

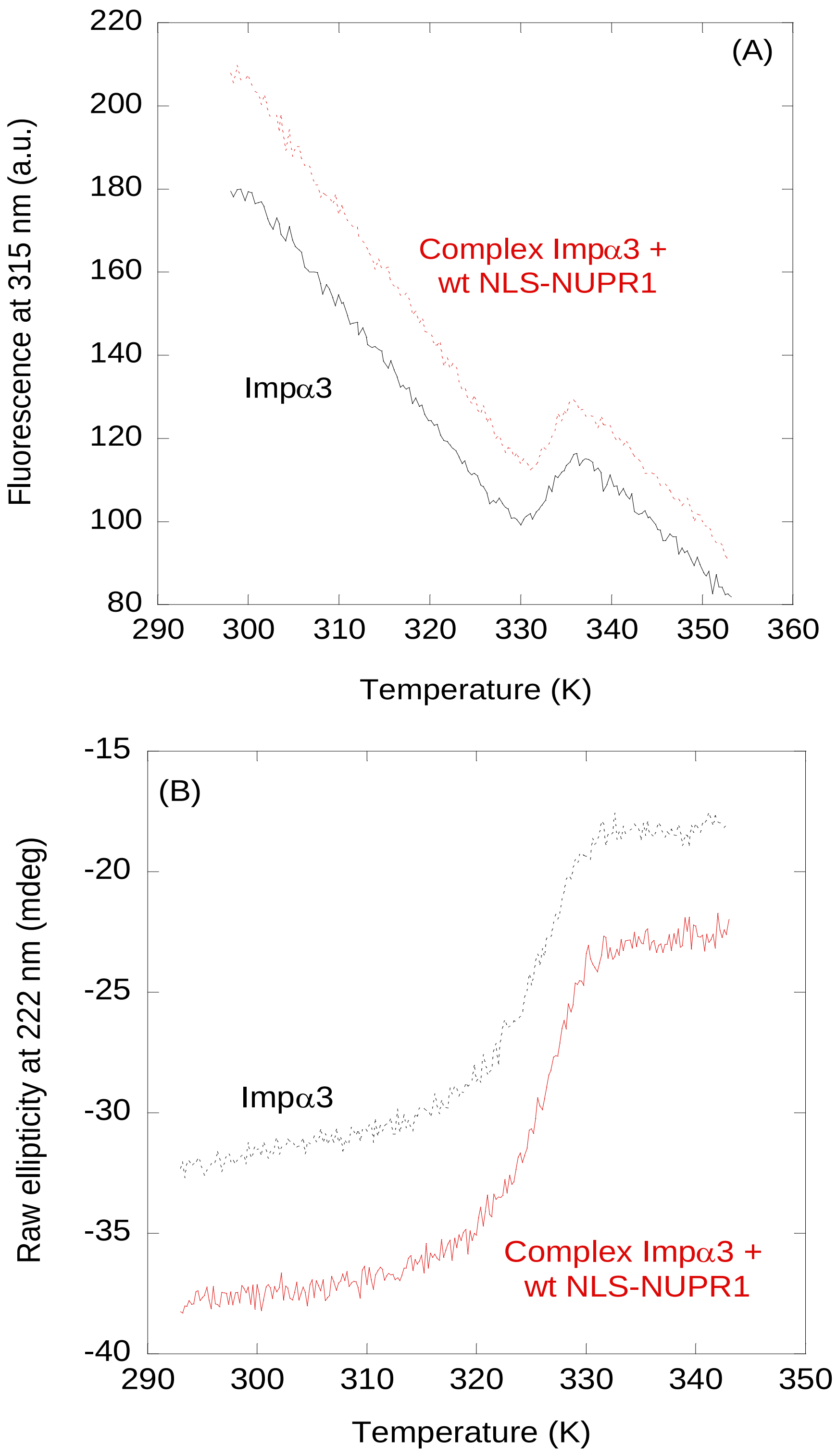

Fig. S6 (Neira et al.)

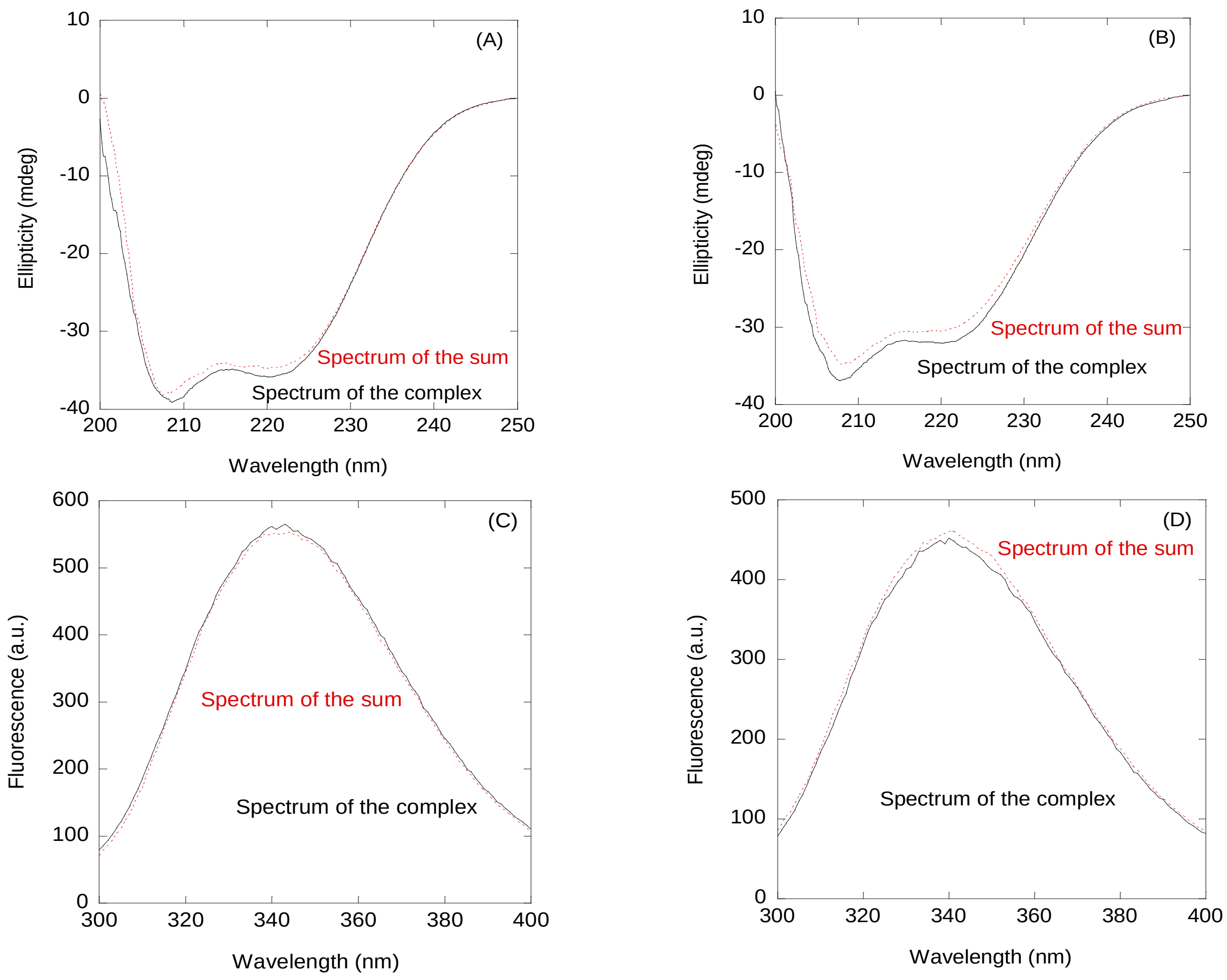

SUPPLEMENTARY TABLES

Table ST1: Chemical shifts (δ, ppm from TSP) of wt peptide in aqueous solution (pH 7.2, 283K)[a]

	NH	Hα	Hβ2	Hβ3	Hγ2	Hγ3	Hδ2	Hδ3	Hε	Hζ
Ac-Ty1	8.36	4.58 (-0.12)	2.99				7.12		6.79	
Thr54	8.11	4.29 (-0.02)	4.13		1.15 (Me)					
Asn55	8.41	4.68 (0.01)	2.76				6.94; 7.66			
Arg56	8.27	4.60 (-0.02)	1.79		1.66		3.15		7.17	
Pro57		4.44 (-0.07)	1.87; 2.00		2.25		3.60	3.78		
Ser58	8.65	4.70 (-0.02)	3.92							
Pro59		4.46 (-0.06)	2.06		2.29		3.72	3.83		
Gly60	8.62	3.86 (-0.09)								
Gly61	8.42	3.88 (-0.09)								
His62	8.51	4.67 (0.02)	3.13; 3.28		8.60 (C2H); 7.25 (C4H)					
Glu63	8.61	4.28 (0.01)	2.00		2.35					
Arg64	8.54	4.27 (-0.02)	1.81		1.60		3.16		7.23	
Lys65	8.57	4.28 (-0.02)	1.75		1.38					
Leu66	8.43	4.35 (-0.03)	1.58				0.88 (Me)			
Val67	8.33	4.17 (0.00)	2.03		0.91 (Me)					

Thr68	8.33	4.33 (-0.02)	4.15	1.19 (Me)	
Lys69	8.44	4.30 (-0.03)	1.80	1.39	
Leu70	8.39	4.33 (0.00)	1.59		0.92 (Me)
Gln71	8.49	4.28 (-0.07)	2.08	2.32	
Asn72	8.56	4.71 (-0.05)	2.81		
Ser73	8.44	4.43 (-0.02)	3.85		
Glu74	8.43	4.38 (0.08)	2.05	2.41	

[a] For the H_α proton column, the value within parenthesis is the conformational shift (δ_{res}-δ_{rc}), where δ_{res} is the chemical shift of such proton for the corresponding residue. The random-coil values for the sequence, δ_{rc}, were obtained from: https://spin.niddk.nih.gov/bax/nmrserver/Poulsen_rc_CS/).

Table ST2: Chemical shifts (δ, ppm from TSP) of K69A peptide in aqueous solution (pH 7.2, 283 K)[a]

	NH	H_{α}	$H_{\beta2}$	$H_{\beta3}$	$H_{\gamma2}$	$H_{\gamma3}$	$H_{\delta2}$	$H_{\delta3}$	H_{ε}	H_{ζ}
Ac-Ty1	8.38	4.59 (-0.11)	2.96				7.13		6.81	
Thr54	8.15	4.31 (0.00)	4.14		1.15 (Me)					
Asn55	8.44	4.63 (-0.04)	2.73				6.94; 7.66			
Arg56	8.31	4.60 (-0.02)	1.79		1.65		3.18		7.20	
Pro57		4.45 (-0.06)	1.87; 2.00		2.26		3.62	3.78		
Ser58	8.66	4.72 (0.00)	3.85							
Pro59		4.42 (-0.10)	2.03		2.29		3.75	3.84		
Gly60	8.59	3.96 (0.01)								
Gly61	8.38	3.90 (-0.07)								
His62	8.55	4.67 (0.02)	3.08; 3.23		8.47 (C2H); 7.24 (C4H)					
Glu63	8.69	4.23 (-0.04)	1.97		2.26					
Arg64	8.54	4.31 (0.02)	1.77		1.64		3.18		7.25	
Lys65	8.59	4.30 (0.00)	1.74		1.42					
Leu66	8.46	4.31 (-0.07)	1.56				0.87 (Me)			
Val67	8.35	4.14 (-0.04)	2.05		0.93 (Me)					
Thr68	8.29	4.32 (-0.02)	4.20		1.18 (Me)					
**Ala69**	8.41	4.29 (-0.05)	1.38 (Me)							

Leu70	8.32	4.27 (-0.04)	1.62		0.90 (Me)
Gln71	8.50	4.31 (-0.04)	1.97; 2.05	2.29	
Asn72	8.59	4.70 (-0.06))	2.81		
Ser73	8.47	4.38 (-0.07)	3.85		
Glu74	8.45	4.31 (0.01)	2.01	2.37	

[a] For the H_α proton column, the value within parenthesis is the conformational shift (δ_{res}-δ_{rc}), where δ_{res} is the chemical shift of such proton for the corresponding residue. The random-coil values for the sequence, δ_{rc}, were obtained from: https://spin.niddk.nih.gov/bax/nmrserver/Poulsen_rc_CS/).

Table ST3: Chemical shifts (δ, ppm from TSP) of pT68 peptide in aqueous solution (pH 7.2, 283 K)[a]

	NH	Hα	Hβ2	Hβ3	Hγ2	Hγ3	Hδ2	Hδ3	Hε	Hζ
Ac-Ty1	8.38	4.56 (-0.14)	3.01				7.13		6.84	
Thr54	8.14	4.27 (-0.04)	4.14		1.15 (Me)					
Asn55	8.43	4.67 (-0.02)	2.78				6.94; 7.66			
Arg56	8.30	4.63 (0.01)	1.79		1.64		3.14		7.20	
Pro57		4.45 (-0.06)	1.87; 2.00		2.25		3.62	3.78		
Ser58	8.66	4.71 (-0.01)	3.82							
Pro59		4.41 (-0.11)	2.01		2.28		3.74	3.83		
Gly60	8.58	3.94 (-0.01)								
Gly61	8.35	3.93 (-0.04)								
His62	8.55	4.72 (0.07)	3.12; 3.25		8.61 (C2H); 7.29 (C4H)					
Glu63	8.69	4.28 (0.01)	2.05		2.30					
Arg64	8.56	4.30 (0.01)	1.75		1.60		3.20		7.25	
Lys65	8.52	4.30 (0.00)	1.76		1.41					
Leu66	8.48	4.30 (-0.08)	1.58				0.87 (Me)			
Val67	8.34	4.14 (-0.03)	2.07		0.92 (Me)					
**pThr68**	8.62	4.40 (0.33)	4.58		1.30 (Me)					
Lys69	8.34	4.33 (0.00)	1.77		1.41					

Leu70	8.34	4.37 (0.00)	1.60		0.91 (Me)
Gln71	8.55	4.28 (-0.07)	2.07	2.33	
Asn72	8.60	4.75 (-0.01)	2.82		
Ser73	8.46	4.41 (-0.04)	3.88		
Glu74	8.49	4.32 (0.02)	2.09	2.34	

[a] For the H_α proton column, the value within parenthesis is the conformational shift (δ_{res}-δ_{rc}), where δ_{res} is the chemical shift of such proton for the corresponding residue. The random-coil values for the sequence, δ_{rc}, were obtained from: https://spin.niddk.nih.gov/bax/nmrserver/Poulsen_rc_CS/). The value for random-coil of the Ha proton of pThr68 was taken to be 4.17 ppm at pH 7.2 [43].

Table ST4: Chemical shifts (δ, ppm from TSP) of T68EK69A peptide in aqueous solution (pH 7.2, 283 K)[a]

	NH	H_{α}	$H_{\beta 2}$	$H_{\beta 3}$	$H_{\gamma 2}$	$H_{\gamma 3}$	$H_{\delta 2}$	$H_{\delta 3}$	H_{ε}	H_{ζ}
Ac-Ty1	8.37	4.58 (-0.12)	2.99				7.13		6.82	
Thr54	8.15	4.30 (-0.01)	4.15		1.13 (Me)					
Asn55	8.44	4.65 (-0.02)	2.77				6.94; 7.66			
Arg56	8.30	4.63 (0.00)	1.82		1.62		3.18		7.20	
Pro57		4.43 (-0.09)	1.86; 2.00		2.28		3.62	3.78		
Ser58	8.67	4.73 (0.01)	3.83							
Pro59		4.46 (-0.07)	1.99		2.33		3.75	3.84		
Gly60	8.59	3.94 (-0.01)								
Gly61	8.37	3.90 (-0.07)								
His62	8.56	4.71 (0.05)	3.16; 3.24		8.61 (C2H); 7.27 (C4H)					
Glu63	8.70	4.24 (-0.03)	1.98		2.28					
Arg64	8.53	4.29 (0.00)	1.78		1.63		3.17		7.26	
Lys65	8.51	4.30 (0.00)	1.69		1.37					
Leu66	8.42	4.32 (-0.07)	1.59				0.88 (Me)			
Val67	8.22	4.04 (-0.05)	2.04		0.93 (Me)					
**Glu68**	8.52	4.27 (0.00)	1.99		2.28					
**Ala69**	8.41	4.27 (-0.03)	1.39 (Me)							

Leu70	8.29	4.31 (-0.01)	1.59		0.90 (Me)
Gln71	8.46	4.31 (-0.04)	2.03	2.32	
Asn72	8.58	4.71 (-0.05)	2.84		
Ser73	8.46	4.39 (-0.06)	3.83		
Glu74	8.47	4.32 (0.03)	2.01	2.33	

[a] For the H_α proton column, the value within parenthesis is the conformational shift (δ_{res}-δ_{rc}), where δ_{res} is the chemical shift of such proton for the corresponding residue. The random-coil values for the sequence, δ_{rc}, were obtained from: https://spin.niddk.nih.gov/bax/nmrserver/Poulsen_rc_CS/).

Table ST5: Chemical shifts (δ, ppm from TSP) of K65AT68E peptide in aqueous solution (pH 7.2, 283 K)[a]

	NH	Hα	Hβ2	Hβ3	Hγ2	Hγ3	Hδ2	Hδ3	Hε	Hζ
Ac-Ty1	8.37	4.58 (-0.12)	2.99				7.13		6.82	
Thr54	8.15	4.30 (-0.01)	4.15		1.13 (Me)					
Asn55	8.44	4.65 (-0.02)	2.77				6.94; 7.66			
Arg56	8.31	4.64 (0.02)	1.80		1.61		3.17		7.20	
Pro57		4.44 (-0.08)	1.86; 1.99		2.28		3.60	3.77		
Ser58	8.67	4.73 (0.01)	3.85							
Pro59		4.44 (-0.09)	2.00		2.31		3.75	3.84		
Gly60	8.60	3.94 (-0.01)								
Gly61	8.37	3.93 (-0.04)								
His62	8.57	4.70 (0.05)	3.15; 3.24		8.61 (C2H); 7.29 (C4H)					
Glu63	8.72	4.25 (-0.03)	2.02		2.31					
Arg64	8.51	4.28 (-0.01)	1.77		1.63		3.20		7.28	
**Ala65**	8.42	4.29 (-0.02)	1.38 (Me)							
Leu66	8.31	4.31 (-0.06)	1.60				0.87 (Me)			
Val67	8.14	4.08 (0.00)	2.03		0.95 (Me)					
**Glu68**	8.50	4.30 (0.04)	2.05		2.27					
Lys69	8.41	4.31 (0.02)	1.80		1.43					

Leu70	8.38	4.28 (-0.05)	1.63		0.88 (Me)
Gln71	8.50	4.28 (-0.07)	2.05	2.33	
Asn72	8.58	4.72 (-0.04)	2.81		
Ser73	8.45	4.39 (-0.06)	3.88		
Glu74	8.51	4.30 (0.01)	2.01	2.36	

[a] For the H_{α} proton column, the value within parenthesis is the conformational shift (δ_{res}-δ_{rc}), where δ_{res} is the chemical shift of such proton for the corresponding residue. The random-coil values for the sequence, δ_{rc}, were obtained from: https://spin.niddk.nih.gov/bax/nmrserver/Poulsen_rc_CS/).

Table ST6: Chemical shifts (δ, ppm from TSP) of K65A peptide in aqueous solution (pH 7.2, 283 K)[a]

	NH	H_{α}	$H_{\beta2}$	$H_{\beta3}$	$H_{\gamma2}$	$H_{\gamma3}$	$H_{\delta2}$	$H_{\delta3}$	H_{ε}	H_{ζ}
Ac-Ty1	8.38	4.61 (-0.09)	2.97; 3.00				7.13		6.84	
Thr54	8.15	4.32 (0.01)	4.18		1.15 (Me)					
Asn55	8.45	4.68 (0.01)	2.78				6.94; 7.66			
Arg56	8.31	4.66 (0.04)	1.83		1.64		3.19		7.21	
Pro57		4.46 (-0.06)	1.89; 1.99		2.24		3.62	3.80		
Ser58	8.67	4.75 (0.03)	3.88							
Pro59		4.45 (-0.08)	2.00		2.32		3.75	3.84		
Gly60	8.61	3.99 (0.04)								
Gly61	8.38	3.95 (-0.02)								
His62	8.47	4.68 (0.02)	3.13; 3.19		8.58 (C2H); 7.27 (C4H)					
Glu63	8.69	4.24 (-0.04)	1.99		2.27					
Arg64	8.51	4.26 (-0.03)	1.84		1.68		3.21		7.26	
**Ala65**	8.39	4.28 (-0.03)	1.39 (Me)							
Leu66	8.43	4.32 (-0.05)	1.60				0.93(Me)			
Val67	8.25	4.18 (0.00)	2.07		0.94 (Me)					
Thr68	8.35	4.31 (-0.04)	4.15		1.19 (Me)					
Lys69	8.43	4.33 (0.00)	1.78		1.41					

Leu70	8.33	4.34 (0.01)	1.64			0.90 (Me)
Gln71	8.51	4.31 (-0.04)	2.01	2.28		
Asn72	8.61	4.73 (-0.03)	2.82			
Ser73	8.46	4.41 (-0.04)	3.89			
Glu74	8.50	4.33 (0.04)	2.01	2.38		

[a] For the H_α proton column, the value within parenthesis is the conformational shift (δ_{res}-δ_{rc}), where δ_{res} is the chemical shift of such proton for the corresponding residue. The random-coil values for the sequence, δ_{rc}, were obtained from: https://spin.niddk.nih.gov/bax/nmrserver/Poulsen_rc_CS/).

Table ST7: Chemical shifts (δ, ppm from TSP) of T68E peptide in aqueous solution (pH 7.2, 283 K)[a]

	NH	H_{α}	$H_{\beta2}$	$H_{\beta3}$	$H_{\gamma2}$	$H_{\gamma3}$	$H_{\delta2}$	$H_{\delta3}$	H_{ε}	H_{ζ}
Ac-Ty1	8.39	4.60 (-0.10)	2.94; 3.00				7.13		6.81	
Thr54	8.15	4.30 (-0.01)	4.15		1.15 (Me)					
Asn55	8.44	4.64 (-0.03)	2.74				6.94; 7.66			
Arg56	8.31	4.64 (0.02)	1.81		1.64		3.16		7.20	
Pro57		4.41 (-0.11)	1.88; 1.99		2.28		3.62	3.79		
Ser58	8.67	4.73 (0.01)	3.82							
Pro59		4.45 (-0.08)	2.02		2.32		3.75	3.84		
Gly60	8.59	3.94 (-0.01)								
Gly61	8.37	3.94 (-0.03)								
His62	8.55	4.69 (0.04)	3.12; 3.23		8.58 (C2H); 7.28 (C4H)					
Glu63	8.71	4.24 (-0.03)	1.95		2.23					
Arg64	8.52	4.30 (0.01)	1.80		1.60		3.18		7.27	
Lys65	8.52	4.29 (-0.01)	1.74		1.40					
Leu66	8.42	4.29 (-0.10)	1.55				0.92 (Me)			
Val67	8.24	4.05 (-0.03)	2.02		0.95 (Me)					
**Glu68**	8.54	4.34 (0.08)	1.98; 2.02		2.28					
Lys69	8.47	4.31 (0.02)	1.74		1.40					

Leu70	8.42	4.29 (-0.04)	1.53		0.92 (Me)
Gln71	8.54	4.27 (-0.07)	2.02	2.28	
Asn72	8.61	4.74 (0.05)	2.83		
Ser73	8.46	4.41 (-0.02)	3.86		
Glu74	8.50	4.29 (0.09)	1.99	2.30	

[a] For the H_α proton column, the value within parenthesis is the conformational shift (δ_{res}-δ_{rc}), where δ_{res} is the chemical shift of such proton for the corresponding residue. The random-coil values for the sequence, δ_{rc}, were obtained from: https://spin.niddk.nih.gov/bax/nmrserver/Poulsen_rc_CS/).

Table ST8: Chemical shifts (δ, ppm from TSP) of K65AT68EK69A peptide in aqueous solution (pH 7.2, 283 K)[a]

	NH	H_α	$H_{\beta 2}$	$H_{\beta 3}$	$H_{\gamma 2}$	$H_{\gamma 3}$	$H_{\delta 2}$	$H_{\delta 3}$	H_ε	H_ζ
Ac-Ty1	8.38	4.63 (-0.07)	2.95; 3.02				7.14		6.84	
Thr54	8.15	4.30 (-0.01)	4.18		1.15 (Me)					
Asn55	8.44	4.63 (-0.04)	2.78; 2.82				6.94; 7.66			
Arg56	8.32	4.66 (0.04)	1.82		1.70		3.18		7.21	
Pro57		4.45 (-0.07)	1.89; 2.01		2.27		3.62	3.80		
Ser58	8.68	4.73 (0.01)	3.82							
Pro59		4.47 (-0.06)	2.05		2.31		3.75	3.85		
Gly60	8.61	3.97 (0.02)								
Gly61	8.37	3.97 (0.00)								
His62	8.59	4.72 (0.07)	3.17; 3.29		8.62 (C2H); 7.31 (C4H)					
Glu63	8.73	4.25 (-0.03)	1.97; 2.06		2.35					
Arg64	8.49	4.30 (0.01)	1.82		1.64		3.21		7.30	
**Ala65**	8.42	4.30 (-0.01)	1.43 (Me)							
Leu66	8.29	4.30 (-0.07)	1.66		1.57		0.93(Me)			
Val67	8.12	4.04 (-0.05)	2.07		0.96 (Me)					
**Glu68**	8.51	4.30 (0.04)	1.95; 2.07		2.36					
**Ala69**	8.36	4.30 (0.00)	1.42 (Me)							

Leu70	8.24	4.30 (-0.02)	1.67	1.59	0.93 (Me)
Gln71	8.42	4.31 (-0.04)	2.04, 2.10	2.39	
Asn72	8.55	4.78 (0.02)	2.87		
Ser73	8.45	4.42 (-0.03)	3.89		
Glu74	8.46	4.31 (0.01)	2.01	2.38	

[a] For the H_α proton column, the value within parenthesis is the conformational shift (δ_{res}-δ_{rc}), where δ_{res} is the chemical shift of such proton for the corresponding residue. The random-coil values for the sequence, δ_{rc}, were obtained from: https://spin.niddk.nih.gov/bax/nmrserver/Poulsen_rc_CS/).
